# Single-trial prediction of reaction time variability from MEG brain activity

**DOI:** 10.1038/srep27416

**Published:** 2016-06-02

**Authors:** Ryu Ohata, Kenji Ogawa, Hiroshi Imamizu

**Affiliations:** 1 Cognitive Mechanisms Laboratories, Advanced Telecommunications Research Institute International (ATR), Keihanna Science City, Kyoto 619-0288, Japan; 2 Graduate School of Frontier Biosciences, Osaka University, Suita, Osaka 565–0871, Japan; 3 Department of Psychology, Graduate School of Humanities and Sociology, The University of Tokyo, Hongo 7-3-1, Bunkyou-ku, 113-0033 Japan; 4 Department of Psychology, Graduate School of Letters, Hokkaido University, Kita 10, Nishi 7, Kita-ku, Sapporo, 060-0810 Japan; 5 Center for Information and Neural Networks (CiNet), National Institute of Information and Communications Technology and Osaka University, Suita, Osaka 565-0871, Japan

## Abstract

Neural activity prior to movement onset contains essential information for predictive assistance for humans using brain-machine-interfaces (BMIs). Even though previous studies successfully predicted different goals for upcoming movements, it is unclear whether non-invasive recording signals contain the information to predict trial-by-trial behavioral variability under the same movement. In this paper, we examined the predictability of subsequent short or long reaction times (RTs) from magnetoencephalography (MEG) signals in a delayed-reach task. The difference in RTs was classified significantly above chance from 550 ms before the go-signal onset using the cortical currents in the premotor cortex. Significantly above-chance classification was performed in the lateral prefrontal and the right inferior parietal cortices at the late stage of the delay period. Thus, inter-trial variability in RTs is predictable information. Our study provides a proof-of-concept of the future development of non-invasive BMIs to prevent delayed movements.

A delayed movement is inevitable regardless how we strive to make a fast reaction. Hence, predicting trial-by-trial variability in subsequent RTs plays a crucial role in preventing accidents due to the delayed reactions of operators. Neurons in the motor-related regions exhibit substantial activity both prior to and during movement execution, and this pre-movement activity is modulated by such parameters as the movement direction of a forthcoming movement^1^,^2^,^3^. Although most previous BMI studies successfully predicted intended goals from neuronal activity for neuroprosthetic control^4^,^5^, it remains unclear whether a delayed reaction can be predicted from pre-movement activity measured by a non-invasive recoding on a single-trial basis.

A number of previous studies have suggested that pre-movement activities in the premotor (PM), the primary motor (M1), the posterior parietal cortex (PPC) and the supplementary motor area (SMA) are involved in motor planning and the preparation for both stimulus-triggered and voluntary movements^6^,^7^,^8^,^9^. Although these areas probably include critical information for predictions, it is unknown whether non-invasive methods can detect subtle differences in neural activity among trials with variable RTs. An electrophysiological study in non-human primates showed the possibility of predicting inter-trial RT variability before movement onset^10^. Afshar *et al.*^10^, whose finding demonstrated that the population dynamics of dorsal PM neurons in monkeys predicted single-trial RTs in delayed-reach tasks, indicated that multi-dimensional activities in PM contain neural information that enables the prediction of variability in subsequent RTs. Therefore we applied a pattern recognition approach, which has been used extensively in functional magnetic resonance imaging (fMRI) research^11^,^12^,^13^,^14^, to MEG signals to predict the differences in RTs before the go-signal onset.

In addition to the influence of preparation activity in the PM cortex, other factors might also cause a delayed movement. Such cognitive states as top-down attention^15^, maintaining an arousal state, and mental effort^16^,^17^ associated with broad brain regions affect variability in behavioral performance. Thus, we hypothesized that the neural activities from several distinct regions over the entire cortical surface other than the PM cortex might represent information that make RTs variable. To test our hypothesis, we examined the prediction performance in each anatomical region to reveal the dominant factors that influence subsequent RTs.

To explore the single-trial predictability of RT variability, we conducted multivariate classification analysis to discriminate between short- and long-RT trials. The cortical currents in separate brain regions were estimated from MEG sensor signals, which were subsequently used as the classifier’s input data. Consequently, differences in RTs were significantly predicted using the cortical activity in the left precentral area: from 550 and 250 ms before the go-signal onset in PM and M1, respectively. Furthermore, the lateral prefrontal and right inferior parietal cortices showed significant classification accuracy of 250 to 0 ms prior to the go-signal onset. These results indicate that preparatory neural activity in PM contains information about variability in RTs even from the early stage of the preparation period. The cognitive factors in the fronto-parietal cortex are also related to subsequent RTs as the movement initiation gets closer. In summary, our study suggests that RT variability is predictable from cortical activity patterns in specific anatomical regions estimated from non-invasive recording signals.

## Results

**Figure 1:**
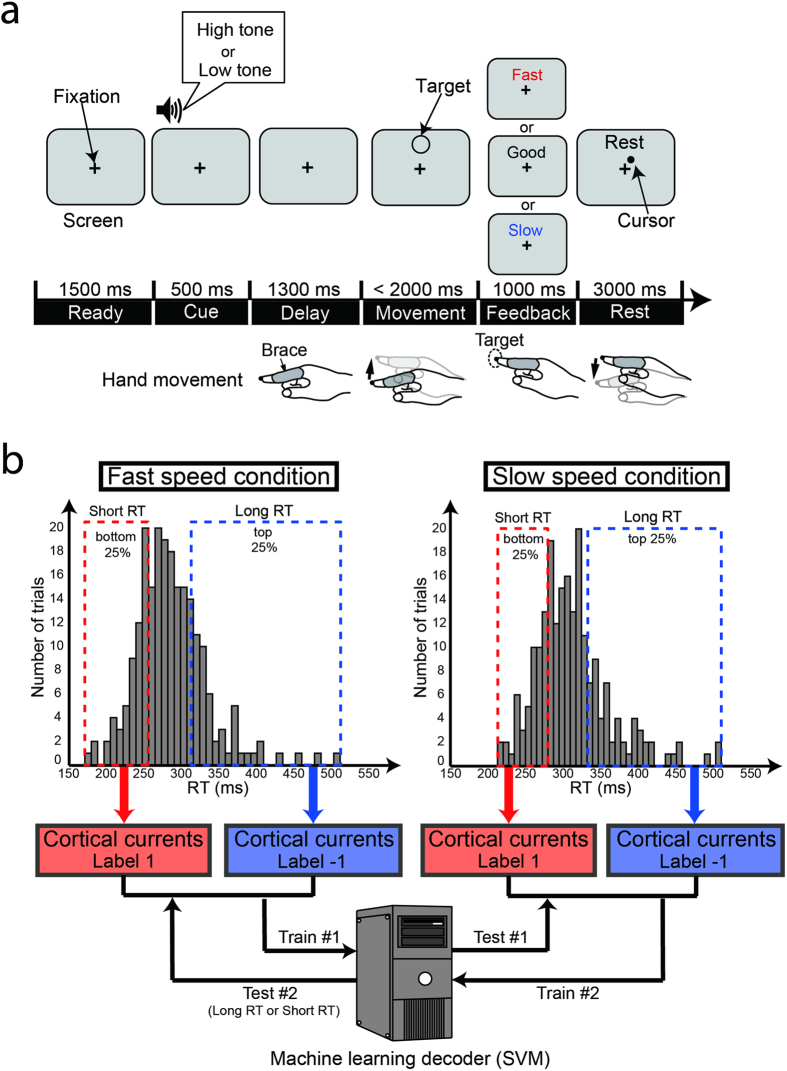
Delayed-finger reach task and classification analysis procedure. (**a**) Experimental paradigm of delayed-finger reach task in MEG experiment. Top panels indicate time series of images viewed by subjects on screen. Bottom figures illustrate postures and movements of their hands corresponding to each period. Subjects initiated finger movements toward a single target as quickly as possible after target presentation (go-signal). (**b**) Schematic illustration of classification analysis procedure. Trials in short- or long-RT groups, defined as bottom and top 25% from the concatenated trials from all sessions, were used for classification analysis. Classifiers were cross-validated using data in different speed conditions. Note that the RT distributions in this figure were obtained from all trials of a single subject (S1). RT ranges in fast-speed condition were 176–260 ms for short-RT group and 315–516 ms for long-RT group. Ranges in slow-speed condition were 217–285 ms for short-RT group and 337–515 ms for long-RT group.

We recorded MEG sensor signals while the subjects conducted a finger-reaching task toward a single target (Fig. 1a). A delay period, during which subjects prepared their upcoming finger movements, separated the Cue period from the target presentation. Subjects were instructed to move their fingers at different speeds (fast or slow) depending on the instruction tones of cue sounds. Our experimental paradigm had the two speed conditions to test the generalizability of a classifier across different speeds (see below) and to enhance the subjects’ concentration on this simple task with variations of the task setting. A session consisted of 60 trials (30 fast- and 30 slow- speed trials). The fast- and slow-speed trials were randomly presented in a session. They were also asked to initiate their finger movements as quickly as possible after the target appearance.

We separately created RT distributions for the fast- and slow-speed conditions (e.g., two distributions in Fig. 1b) for individual subjects. We concatenated the trials from all the sessions for creating the distributions. A short- or long-RT label (1 or −1) was attached to the trials belonging to the top or bottom 25% of the RT distribution, respectively. We trained a support vector machine (SVM) classifier to distinguish the long- or short-RT trials using the trials of one speed condition, and the classifier was tested with the trials of the other speed condition (two-fold leave-one-out cross-validation). This procedure enables us to test the generalizability of the classifiers across different speed conditions and to investigate factors for RT variability common to different movement conditions. The classification analysis was performed within subjects. The mean numbers of the trials used for classifier training were 98 (SD: 16) and 95 (18) in the fast- and slow-speed conditions, respectively. We evaluated the prediction performance to investigate whether the classification was significantly above chance using cortical currents prior to the go-signal onset.

### Behavior results

We defined RT as the length of time from the target appearance to the movement onset. The mean RTs across subjects were 450.6 ms (SD: 69.6) for all the trials, 458.0 ms (81.6) in the fast-speed condition, and 443.5 ms (61.0) in the slow-speed condition. Note that no significant difference was identified between the two conditions across subjects (two-tailed *t*-test; *t*_(14)_ = 0.40, *p* = 0.69). For individual subjects, four out of eight showed significant differences in RTs in the two speed conditions (Supplementary Fig. S1a). RTs in the fast-speed condition were significantly shorter than those in the slow-speed condition in one of the four subjects, and the opposite pattern was observed in three subjects (see legend of Supplementary Fig. S1 for statistical details). This result indicates that the conditions had no consistent effect on the RTs. On the other hand, every subject showed a significantly higher maximum fingertip’s tangential velocity in the fast-speed condition than the slow-speed condition (Supplementary Fig. S1b). This result suggests that all subjects correctly applied the speed instructions in the two conditions.

### Cortical current estimation

**Figure 2:**
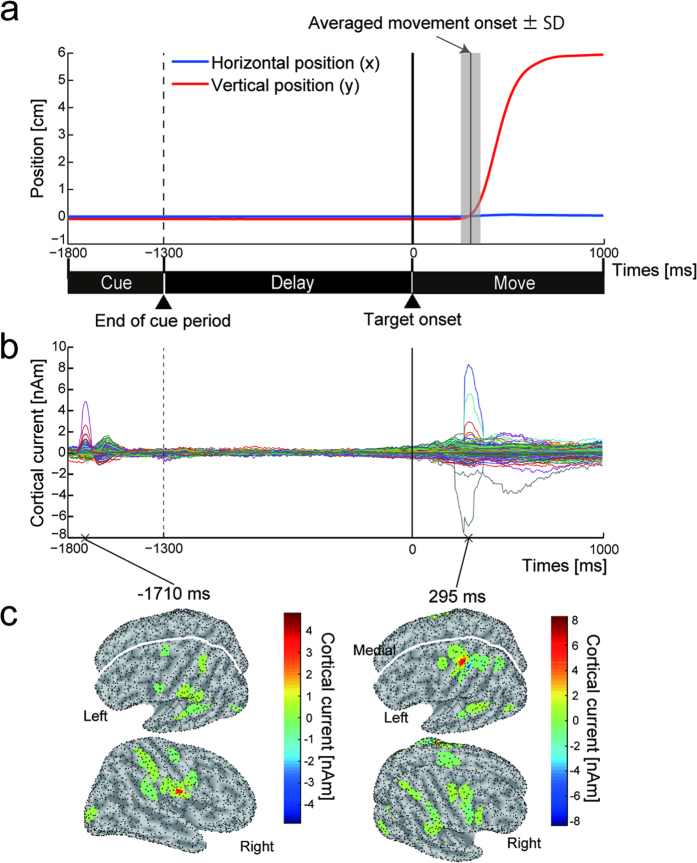
Temporal dynamics of cortical currents for delayed-finger reach task. (**a**) Time courses of horizontal (blue) and vertical (red) positions of fingertip when a subject (S1) moved it toward target. Vertical solid line in gray shaded area denotes averaged movement onset ± SD (304.0 ± 50.9 ms). (**b**) Time courses of cortical currents estimated by a hierarchical Bayesian method. One colored line represents cortical current from one source dipole. Time courses are aligned to target onset (0 ms) and averaged across trials and sessions. Vertical dashed and solid lines indicate end of cue period and target onset timing, respectively. (**c**) Absolute value of cortical currents rendered on inflated cortical surfaces sampled at 1710 ms before and 295 ms after target onset (corresponding to panel **b**). Black dots on surface denote vertices at which current dipoles were estimated.

We estimated 3,197 ± 210 (mean ± SD across subjects) single-current dipoles on the cortical surface. An inverse problem (projection from sensors to current sources) was solved by a hierarchical Bayesian method using fMRI activity (one subject’s data in Supplementary Fig. S2) as a hierarchical prior^18^,^19^. Figure 2a shows the vertical and horizontal trajectories of the fingertip position for a single subject during the Cue, Delay and Move periods. Figure 2b shows the time courses of the estimated cortical currents of the dipoles (3,439 single-current dipoles for the subject) on the same timeline as the finger trajectory (Fig. 2a). They were aligned to the target onset (0 ms) and averaged across trials and sessions. Prominent changes in the currents were observed within the Cue (positive peak at −1710 ms) and Move periods (positive peak at 295 ms). The maximum values of the peaks were detected at the dipoles in the right auditory cortex for the first peak and in the left primary motor cortex for the second peak (Fig. 2c). This result shows that the cortical currents related to this task’s execution were reliably estimated.

### Prediction of RT differences

**Figure 3:**
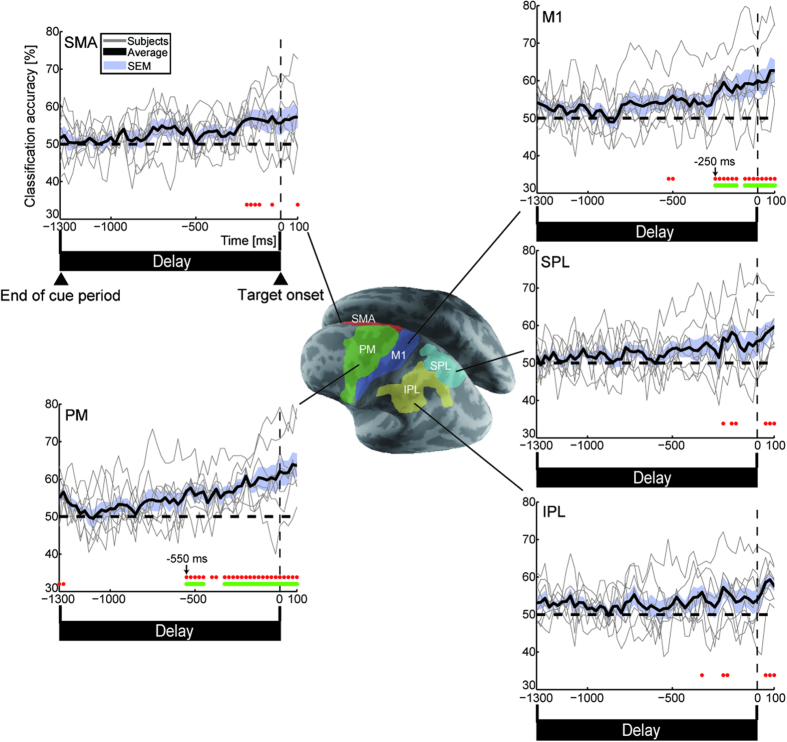
Classification performance in functionally selected areas. Time courses of classification accuracies in left PM, M1, SMA, SPL and IPL from start of Delay period (−1300 ms) to 100 ms after target onset. Accuracies for individual subjects (thin gray solid lines) were averaged over all subjects (thick black solid lines). Blue shaded area denotes SEM across subjects. Rows of red dots indicate significant time points (*n* = 8, both two-tailed *t*-test *p* < 0.05 and group-level permutation test *p* < 1 × 10^−4^), and green horizontal lines show consecutively significant time points corrected by a time-cluster-based approach.

To explore the possibility of predicting RT variability, we first conducted classification analysis using the cortical activity in five regions that are functionally related to motor planning and preparation: PM, M1, SMA, the superior parietal lobule (SPL), and the inferior parietal lobule (IPL) in the left (contralateral to the hand being used) hemisphere (Fig. 3). We employed a sliding time window to investigate whether the classification performance depends on the time to the go-signal onset^20^,^21^,^22^. Cortical currents were divided into 100-ms time windows with 25% overlap (25-ms steps). The classifiers were trained and tested using cortical currents that were temporally averaged within each time window. For all of the time windows, we determined the significance by both a two-tailed *t*-test and a group-level permutation test (see Methods). For multiple comparison corrections, a time-cluster-based approach was applied in which a cluster of time points was significant after the correction only when it had more than five consecutively significant time points^23^ (see Supplementary Methods: Time-cluster-based approach for multiple comparison correction). The rows of red dots and green horizontal lines below each panel in Fig. 3 indicate the time at which the classification accuracy significantly exceeded chance without and with multiple comparison correction, respectively. Consecutively significant time points were first observed at 550 ms (time window: 500–600 ms) and 250 ms (200–300 ms) before the target onset in PM and M1, respectively. In contrast, the classification performance in the three other regions did not reach a significant level before the go-signal onset.

Regarding the classification performance for individual subjects, we investigated significant time points above chance using a binomial test (*p* < 0.05) with a time-cluster-based approach (Supplementary Fig. S3). Although we obtained consecutively significant classification in PM during the delay period for seven out of eight subjects, its onsets were variable across subjects. The following were the onset timings for PM: Subject 1 (S1); −400 ms, S2; no consecutively significant time points during the delay, S3; −250 ms, S4; −725 ms, S5; −1300 ms, S6; −575 ms, S7; −225 ms, S8; −50 ms. A possible reason for this variability in the onsets is that the information for discriminating differences in subsequent RTs was not time-locked during the delay period. To quantitatively evaluate the variability in the onsets across subjects, we estimated 95% confidence intervals for the onset with a bootstrap method^21^,^24^ (see Supplementary Methods: 95% confidence interval estimation of onset of significant classification by a bootstrap method). The following confidence intervals were found for the five regions: PM: −589 to −315 ms; M1: −380 to −46 ms; SMA: −356 to 107 ms; SPL: −90 to 154 ms; and IPL: −113 to 107 ms. Note that the confidence intervals for PM and M1 did not exceed the go-signal timing. These results suggest that the classification performances for PM and M1 were certainly above chance during the delay period.

To confirm that our significant results were not due to factors unrelated to RT variability, we conducted three additional analyses. First, we assessed the influence of session-by-session difference on the classification results. This was done because we allocated a different number of trials to short- and long-RT labels in different sessions in order to define each RT group as the top or bottom 25% of the RT distributions among the trials concatenated from all sessions. To exclude this influence, we incorporated a leave-one-session-out cross-validation procedure in our classification method (see Supplementary Methods: Classification method based on a leave-one-session-out cross-validation procedure). The classifier was trained using the trials in one speed condition except for one session’s data and then tested using the left-out session’s data in the other speed condition (Supplementary Fig. S10). This procedure was repeated until all sessions’ data in each speed condition became test data. If the session-difference were a critical source of information for the classification in our previous analysis, this procedure would not obtain a significant classification performance. However, significant time points were obtained from 250 ms and 225 ms before the go-signal for PM and M1, respectively (Supplementary Fig. S4). This result confirmed that RT variability could be predicted even when excluding session-by-session differences in the number of short- and long-RT trials.

As a second issue for analysis, the number of single-current dipoles was different among the five motor-related regions (Supplementary Table S1). To examine whether the difference in the number of dipoles influenced classification performance among the regions, we conducted the same classification procedure by equalizing the number of dipoles across the regions. We randomly selected dipoles in each motor-related region at a number equal to that of the region with the smallest number of dipoles within individual subjects. For example, we used 26 dipoles randomly selected in each region for Subject 1 data because the smallest number of dipoles in a single region was 26 (SMA; see Supplementary Table S1) among the five regions. We repeatedly conducted the selection and classification procedures 100 times to obtain accuracy that was less biased by random selection. Consequently, the onsets of consecutively significant classification for PM and M1 were observed at 250 ms and 225 ms before the go-signal, respectively (Supplementary Fig. S5). The classification accuracy in the other three regions did not reach the significant level before the go-signal onset, similarly to the results without equalization of the number of dipoles. PM and M1 showed above-chance accuracy before the go-signal onset even if the number of dipoles were equalized, suggesting that the activity in PM and M1 certainly contains the predictive information of RT variability within the motor-related regions.

Finally, to confirm that our successful classification of cortical currents was not due to a systematic artifact caused by the estimation of cortical currents from the MEG sensor signals, we conducted RT classification using the MEG signals of all sensors as input data. The classification accuracy significantly exceeded chance from 350 ms before the target onset (Supplementary Fig. S6). Thus, even the sensor signals, from which the cortical currents were estimated, have information to predict RT variability before the go-signal onset. That is, the above-chance accuracy using the cortical currents was not due to the artifacts yielded by the source current estimation.

### Classification performance in separate brain regions of whole brain

**Figure 4:**
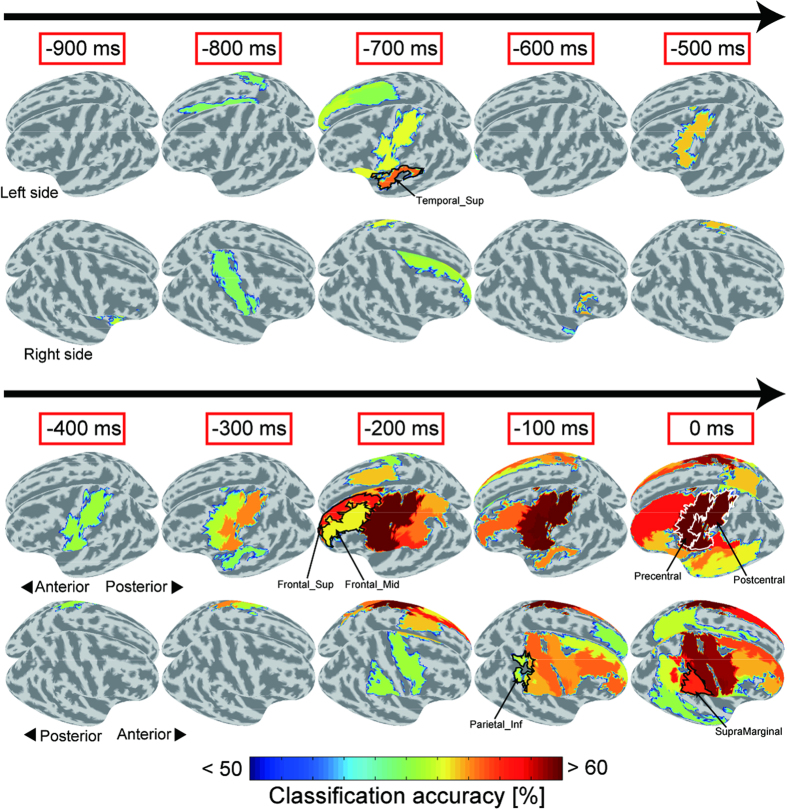
Classification accuracies in separate regions over entire cortical surface. Averaged classification accuracies were calculated in 80 AAL map regions and overlaid on cortical surface from 900 to 0 ms before target onset in 100-ms steps. Regions were colored only when their classification accuracies were significantly above chance (*n* = 8, both two-tailed *t*-test *p* < 0.05 and group-level permutation test *p* < 1 × 10^−4^). Arrows denote flow of time during delay period. Upper panel in each time window shows upper-left side of cerebral hemispheres while lower panel shows upper-right side.

Next, we explored the areas that show significant prediction performance before the go-signal onset among the anatomical regions of the whole brain. We conducted classification analysis using the cortical currents within 80 individual ROIs defined by the automated anatomical labeling (AAL) atlas^25^. Figure 4 shows the classification accuracies averaged across subjects, color-coded and overlaid on the inflated cortical surface from 900 to 0 ms prior to the target onset in 100-ms steps. The figure shows only those regions in which the classification accuracies were significantly above chance. The accuracy in the left central area, consisting of pre and postcentral gyri (enclosed by the white curve in Fig. 4, see left hemisphere at 0 ms), showed a significant level from 500 ms before the target onset. High accuracies were also observed in several other regions. We investigated the time courses of the classification accuracy for each AAL map region and consecutively significant above-chance classification during the delay periods (Table 1). The lateral prefrontal cortex (Frontal_Sup_L/R, Frontal_Mid_L/R) and the right inferior parietal cortex (Parietal_Inf_R, SupraMarginal_R) showed continuous above-chance accuracy from the late stage of the delay period (the earliest onset was 250 ms before the target appearance for Frontal_Sup_L). In contrast, we obtained significant accuracy in the left superior temporal area (Temporal_Sup_L) at the early stage of the delay period, which was between −725 and −625 ms. These results indicate that various regions other than those related to motor planning/preparation influence trial-by-trial variable RTs. Note that due to the different number of dipoles estimated in different ROIs, we were not able to compare the classification accuracy among these regions; however, our purpose was to explore regions from which the variability of RT can be predicted before the go-signal.

## Discussion

Neurophysiological studies suggest that neural activity during the preparatory period is a crucial source of behavioral variability^10^,^26^,^27^. Our MEG study demonstrates that pre-movement signals contain significant information to predict whether long or short RTs occur in the forthcoming movements. To the best of our knowledge, no study has successfully predicted RT variability across trials from non-invasive recording signals. A classifier trained by using the cortical currents in PM, which is observed to be mainly correlated with trial-by-trial RT variability in primate studies^10^,^27^, exhibited significant above-chance performance from 550 ms before the go-signal onset (Fig. 3). In addition, activity in the lateral prefrontal and right inferior parietal cortices yielded high classification accuracy from the late stage of the preparation period (Fig. 4 and Table 1). Thus, MEG signals covering the entire cortical surface provide useful information for exploring the relationship between pre-movement neural activities and subsequent RTs over a wide range of brain areas.

Trial-by-trial neural variability in the left precentral area, consisting of PM and M1, is the dominant factor for the differences in RTs (Fig. 3). Neurons not only in PM but also in the parietal region encode the planned movements, and the recorded activities were utilized to control neuroprosthetic devices^28^. However, the prediction performance in the left parietal regions did not reach a significant level during the delay period (Fig. 3). This result suggests that since the neurons code the goals of the intended movements, not all of them can represent the movement onset variability. Our result is consistent with a recent neurophysiological study. Michaels *et al.*^27^ demonstrated that activity in the macaque premotor cortex (hand area: F5) explained a larger portion of the variability in RTs than in the parietal cortex (anterior intraparietal area) during a delayed grasping task^27^. Even though both regions are involved in planning the grasping movement, only the pre-movement activity in the premotor cortex dominantly affects the trial-by-trial variability in RTs.

The right inferior parietal cortex showed significant above-chance performance immediately before the go-signals (Fig. 4 and Table 1). Pre-movement activity in the inferior parietal cortex is involved in the conscious intention to move as well as the motor preparation process^29^. Robust motor intention cannot always be maintained during the delay period in every trial. Our study suggests that the ready to move mental state (conscious intention) is another factor that affects reaction times.

Neural activity related to motor preparation isn’t the only factor that predicts differences in subsequent RTs. Both sides of the lateral prefrontal cortex also contributed to predictions in the late stage of the delay period, which is 250 to 0 ms before the go-signal onset (Fig. 4 and Table 1). Many studies suggest that the prefrontal and parietal cortices are the essential components of top-down attentional networks^30^,^31^. In particular, the involvement of fronto-parietal activity in sustained attention during the delay period is essential for quick reactions. Meta-analysis of neuroimaging studies suggested that the right lateral prefrontal cortex and the inferior part of the parietal areas (the intraparietal sulcus and the temporoparietal junction) promote the sustained attention required for vigilance tasks^32^. In addition, persistent activity in the lateral prefrontal cortex was found in delayed motor tasks, suggesting that it is involved in the working memory that stores upcoming motor plans^33^,^34^,^35^. Although the target was presented in only one direction, subjects had to prepare two movement speeds in our experimental paradigm. Retaining appropriate motor plans during delay periods is one influential factor of RT variability. Thus, top-down control in the fronto-parietal cortex relevant to sustained attention and the maintenance of motor plans are critical clues to predict delayed responses.

Although some studies applied multivariate pattern classification to MEG signals to obtain the temporal dynamics of neural representation^20^,^21^,^22^,^24^, few have used cortical currents after solving the inverse problem as the classifier’s input data. Our current estimation method successfully separated the cortical currents from a mixture of MEG sensor signals originating from many regions for precise anatomical segmentation of the current sources. This separation allowed us to investigate the classification accuracies calculated from the cortical current patterns within separate brain regions. The effectiveness of using estimated cortical currents in previous studies improved the estimation accuracy for the reconstruction of computer cursor movements^36^, finger movements^37^ and muscle activities^38^.

Two of our additional analyses described below suggest that a multivariate pattern of single-current dipoles, which is the relative variation in the source current value, contains the information for the discrimination of short and long RTs. First, to investigate the characteristics of PM currents that allow the classifier to make a correct prediction, we compared the within-subject cortical currents averaged across the dipoles in PM for short vs. long RTs (Supplementary Fig. S7). The current values in several subjects were significantly different between the short- and long-RT groups during the delay period (two-tailed *t*-test; *p* < 0.05). However, the classifier, which was trained using the currents at which we identified significant differences in the current values, did not always achieve above-chance levels (binomial test; *p* < 0.05). This fact suggests that the magnitude of the spatially averaged activity is not the main factor that enables us to predict RT variability.

To examine whether specific regions in PM are associated with short or long RTs, we investigated the dipole bias map that displayed either positive or negative signs of the product of the weights and the currents averaged across the training data (Supplementary Fig. S8). Note that the training data have the same number of trials for short as for long RTs when calculating the dipole bias map. A map was made for each subject. The sign of the product represents the bias of each dipole toward short- or long-RT groups; a dipole with a positive sign contributes to the classification of a trial as a short-RT group, while a dipole with a negative sign contributes to the classification of a trial as a long-RT group. We used the weights and currents at 50 ms before the go-signal onset when significantly above-chance accuracy (binomial test: *p* < 0.05) was obtained in most of the subjects (six out of eight; see PM panel in Supplementary Fig. S3). Consequently, we observed intermingled patterns in every subject, which supports the idea that the information was hidden in a complex pattern of cortical currents.

For future BMI applications, we have to solve at least three major problems before implementing an online prediction system to monitor brain states for warnings about delayed responses. First, we must improve the prediction performance because the classification accuracy was not very high (62.0% at the go-signal onset for PM). Second, we must take into account the variable onsets of consecutively significant classifications across subjects. This fact might require the development of a prescreening system to limit users to people whose RTs are accurately predicted from the early stage of the preparation period. Finally, our method must be tested with a portable measurement system, such as electroencephalography. However, our study provides a proof-of-concept of the future development of non-invasive BMIs to prevent delayed movements.

In summary, trial-by-trial variability in RTs can be predicted from the early stage of the preparation period. We showed that not only preparatory activity in the motor cortex but also the cognitive and conscious states in the fronto-parietal regions probably influence subsequent RTs. Although most previous BMI studies assumed that motor information must be decoded as a control signal for prosthetic devices^4^,^5^,^39^,^40^, our results indicate that utilizing pre-movement non-invasive neural activity might prevent delayed reactions that often lead to serious human error^16^,^17^.

## Methods

### Participants

Nine right-handed males (22–45 years of age) participated in our experiment. All participants gave informed written consent. The study was approved by the Ethics Committee at Advanced Telecommunication Research Institute International (www.atr.jp), and the experimental protocol was carried out in accordance with the latest version of the Declaration of Helsinki.

### Task procedure

The subjects moved their right index fingers toward a target in a delayed-reach task. Their forearms were fixed to a bed, and their finger joints were immobilized by a brace that only allowed wrist movements. A motion tracking marker of a position recording system (Radish 3D; Library Inc., Japan) was attached to their right index fingertip. The marker position was recorded at a sampling frequency of 60 Hz. The sampling rate was increased to 1 kHz using spline interpolation to equalize the rate of the sampled MEG data. RT was defined as the time length from the target appearance to the movement onset, which was set to the first time at which the fingertip’s tangential velocity exceeded 5% of the maximum velocity.

The trials began with a screen on which only a fixation cross was visible (timeline in Fig. 1a). After a 1500-ms Ready period, a high (880 Hz) or a low (440 Hz) instruction tone was presented for 500 ms (Cue period). The high or low tones indicated the fast- or slow-speed movements required in subsequent movements. Correspondence between the tone and speed conditions was counterbalanced across subjects. A target appeared above the fixation cross after a 1300-ms Delay period. Subjects initiated a finger movement as quickly as possible within the next 2000-ms Movement period. 200 ms after reaching the fingertip at the target circle, a feedback instruction (good, fast, or slow based on finger movement speed; see “Feedback instruction” in Supplementary Methods) was presented for a 1000-ms Feedback period. They returned the cursor to the starting position and kept it within the next 3000-ms Rest period. A session consisted of 60 trials (30 fast- and 30 slow-speed trials). Fast- and slow-speed trials were randomly presented in a session. Subjects underwent six to nine sessions in the MEG experiment. We simultaneously recorded electrooculography (EOG) to detect blinks and eye movements. Eye movements (including blinks) were not allowed from the beginning of the Cue periods to the end of the Feedback periods.

The fMRI sessions were composed of alternating blocks of execution (21.2 s) and rest periods (15 s). In the former, subjects conducted four trials, all of which were identical to those in the MEG experiment except that the movement period was fixed to 1.5 s. In the latter, Rest was presented on the screen for the first 2 s, and subjects fixed their eyes on the fixation cross that was presented for the remaining 13 s without finger movements. Each subject performed two sessions of fifteen blocks.

### MEG data acquisition and preprocessing

A whole-head 400-channel system (PQ1400RM; Yokogawa Electric Co., Japan), which consists of a 210-channel axial gradiometer and a 190-channel planar gradiometer, was used for MEG recording at a sampling rate of 1 kHz. Only the axial gradiometer data were used in the analyses. We subtracted the environmental noise estimated from the reference magnetometer signals from the neurophysiological signals using time-shift Principled Component Analysis^41^. The signals were then band-pass-filtered from 0.1 to 90 Hz and downsampled at 200 Hz. For each trial, the signal value was adjusted so that the mean value from 2000 to 1800 ms before the target onset became zero (baseline interval) because the influence of the activity evoked by the finger movements is the lowest immediately before the Cue period in comparison to the other periods. Trials and sensors were excluded from further analysis according to the trial and sensor rejection criteria (see “Trial and sensor rejection criteria” in Supplementary Methods). One subject was excluded from analysis due to an insufficient number of trials (fewer than 50% of the total trials) after rejection of the noisy trials. Thus, the mean number of the remaining trials and sensors were 386 (SD: 66) and 206 (SD: 9.6), respectively.

### fMRI data acquisition and preprocessing

We used a 3 Tesla Magnetom Verio scanner (Siemens, Germany) to obtain the blood oxygen level-dependent (BOLD) contrast function images. The images, weighted with the apparent transverse relaxation times, were obtained with an echo planar imaging (EPI) sequence. 190 scans were acquired in each session with a gradient echo EPI sequence under the following scanning parameters: repetition time, 3 s; echo time, 30 ms; flip angle, 80°; voxel size, 3 × 3 × 3 mm; matrix, 64 × 64 mm; 47 axial slices; and thickness, 3 mm without gaps. T1-weighted structure images were obtained with 1 × 1 × 1 mm resolution with a gradient echo sequence (repetition time, 2250 ms; echo time, 3.06 ms; flip angle, 9°; matrix, 256 × 256; 208 axial slices; and thickness, 1 mm without gap).

The fMRI data were analyzed using SPM8 (Wellcome Trust Centre for Neuroimaging, London, UCL) on Matlab. We discarded the first three volumes of the images in each session to allow for T1 equilibration. The remaining image volumes were realigned to the first image to correct for head movements. The data were spatially normalized to the Montreal Neurological Institute (MNI) (Montreal, Quebec, Canada) reference brain and resliced to a 2-mm isotopic voxel size. The data were smoothed spatially with a 6-mm full-width at half-maximum Gaussian kernel. The voxel time series were high-pass filtered with a cutoff frequency of 128 s to remove slowly varying trends.

Statistical parametric maps of *t* statistics were calculated for each subject. The box-car functions were convolved with a canonical hemodynamic response function in SPM8 to yield regressors in a general linear model. We used SPM contrast to compare the estimated parameters (execution - rest) to yield *t*-maps (*p* < 0.001, uncorrected).

### Cortical current estimation using a hierarchical Bayesian method

To map the current dipoles on the cortical surface, we constructed a polygon model of the cortical surface based on MR structure images using FreeSurfer software^42^. Based on previous studies^37^,^38^, two types of parameters, a variance magnification parameter (m0) and a confidence parameter (γ0), were set at 100 and 10 for the estimation using fMRI prior information^18^,^19^. In the estimation, we incorporated the artifact dipoles that were located at the center of the heart and the carotid arteries to remove the effect of artifacts by heart beats, the right shoulder and wrist joints to discard artifacts by muscle activities caused by finger movements, and the left and right eyeballs to remove artifacts by eye movements^43^,^44^.

Inverse filters were estimated for 59 divided time windows (100-ms long, 50-ms overlap) from 2 s before to 1 s after timing the target appearance. We separately calculated an inverse filter for each time window. In the overlap periods, they were concatenated sequentially.

### Multivariate pattern classification on MEG data

Short- or long-RT trials were classified using a linear SVM implemented in LIBSVM (http://www.csie.ntu.edu.tw/~cjlin/libsvm/) with default parameters (a fixed regularization parameter C = 1)^45^. For individual subjects, we separately concatenated the trials of all sessions for the two speed conditions and defined the short- and long-RT classes as the bottom 25% and the top 25% from the concatenated trials in each speed condition. The SVM classifier was trained to discriminate the short- or long-RT-group trials in the fast-speed condition and tested in the slow-speed condition and vice versa (Fig. 1b).

To investigate the time course of the classification accuracy, we trained a new classifier and tested it for each 100-ms time window with 25% overlap (sliding time window decoding). The cortical currents within each time window were temporally averaged. We evaluated the statistical significance for classification accuracy by combining the parametric and nonparametric methods. First, we conducted two-tailed *t*-tests to determine whether the classification accuracy significantly exceeded chance level (50%) with a threshold of *p* < 0.05 at each time point. Second, we conducted a group-level permutation test^46^. The classification accuracy was computed with the surrogate data in which the relationship between the labels and the data was shuffled. After repeating this procedure 100 times for each subject, we randomly sampled 10^4^ combinations of the data of eight subjects. We then averaged the classification accuracy for each combination to create an empirical distribution and investigated whether the classification accuracy with correct labeling exceeded the 9,999th percentile of the distribution (i.e., *p* = 1 × 10^−4^). This threshold followed a previous study^46^. The mean threshold (classification accuracy) for the PM averaged across the time points during the delay was 55.3%. For multiple comparison corrections, we applied a time-cluster-based approach. A time point was considered significant only when it was a member of a cluster of at least five consecutively significant time points (see Supplementary Methods).

### Definitions of regions of interest (ROIs)

First, we examined the classification performance in the five functionally selected areas: PM, M1, SMA, SPL, and IPL. These areas were anatomically defined with the AAL atlas^25^ and the Brodmann areas that are included in the WFU PickAtlas^47^. PM and M1 were defined as Broadmann area 6 excluding the SMA of the AAL atlas and Broadmann area 4. Three other areas were determined by AAL (Supp_Motor_Area_L, Parietal_Sup_L, and Parietal_Inf_L). Furthermore, we defined 80 ROIs based on the AAL atlas in the cortical surface and examined the classification accuracies in each ROI.

## Additional Information

**How to cite this article**: Ohata, R. *et al.* Single-trial prediction of reaction time variability from MEG brain activity. *Sci. Rep.* **6**, 27416; doi: 10.1038/srep27416 (2016).

## Supplementary Material

Supplementary Information

## Figures and Tables

**Table 1:** AAL areas, number of vertices with SD, and consecutive time points exhibiting significant above-chance accuracies.

Region	Number of vertices (SD)	Consecutively significant time points
L Precentral	48 (7.6)	−550~−450, −275~0
R Precentral	46 (8.7)	−175~0
L Postcentral	60 (6.8)	−325~0
L Frontal Sup	49 (6.1)	−250~−150
R Frontal Sup	58 (3.7)	−100~0
L Frontal Mid	69 (10)	−100~0
R Frontal Mid	80 (12)	−175~0
R Parietal Inf	21 (5.3)	−100~0
R SupraMarginal	32 (8.5)	−100~0
L Temporal Sup	32 (6.7)	−725~−625, −125~0
