# Supplementary material for: Single-trial prediction of reaction time variability from MEG brain activity
Source: Sci Rep. 2016 Jun 2;6:27416. doi: 10.1038/srep27416 (PMC4889999; doi:10.1038/srep27416)
Supplement: Supplementary Information [file srep27416-s1.pdf]

Supplementary Information

Single-trial prediction of reaction time variability from MEG brain activity

Ryu Ohata, Kenji Ogawa, and Hiroshi Imamizu

Correspondence to H.I (imamizu@gmail.com) or R.O (ryu.oohata@gmail.com)

SI Figures

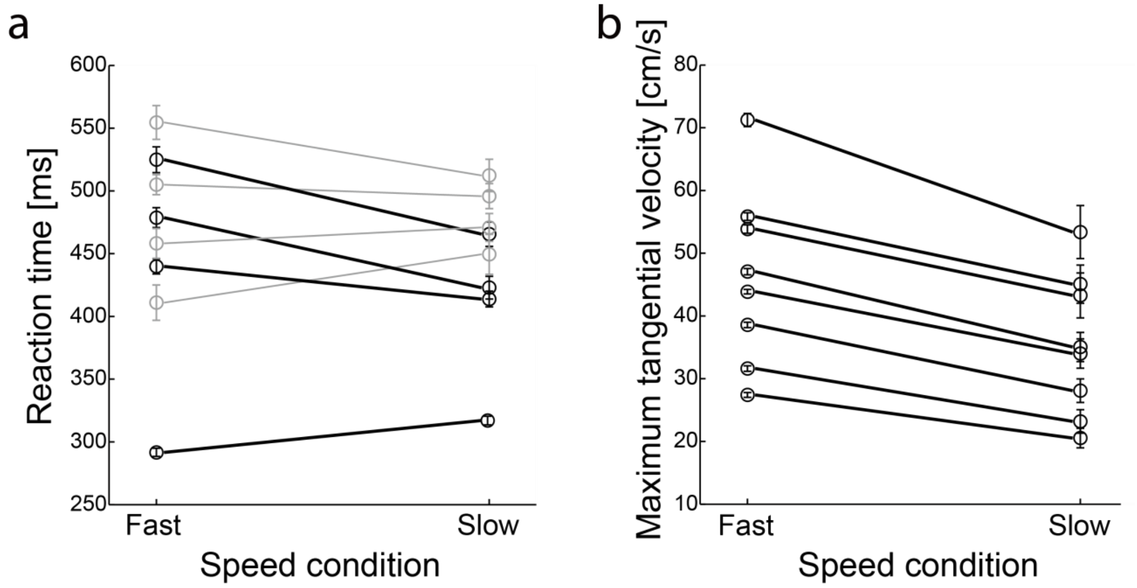

Supplementary Figure S1 | Behavioral results in fast- and slow-speed conditions. (a) RTs averaged across all trials for every subject. Each line corresponds to a subject. Black and gray lines indicate subjects whose RTs were significantly and non-significantly different between the two speed conditions, respectively (two-tailed *t*-test separately applied to individual data; $p < 0.05$, corrected by Bonferroni for multiple comparisons). (b) Maximum tangential velocity of fingertips averaged across all trials. Velocity in fast-speed condition was significantly higher than that in slow-speed condition in all subjects (two-tailed *t*-test; $p < 0.05$, corrected by Bonferroni for multiple comparisons). Error bars indicate standard error of mean.

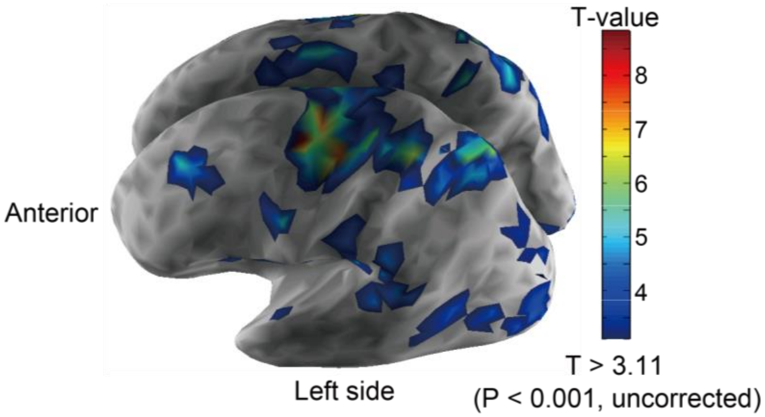

Supplementary Figure S2 | fMRI activity while one subject conducted delayed-finger reach task. *T*-value was calculated to contrast activity in execution periods to that in rest periods ($p < 0.001$, uncorrected).

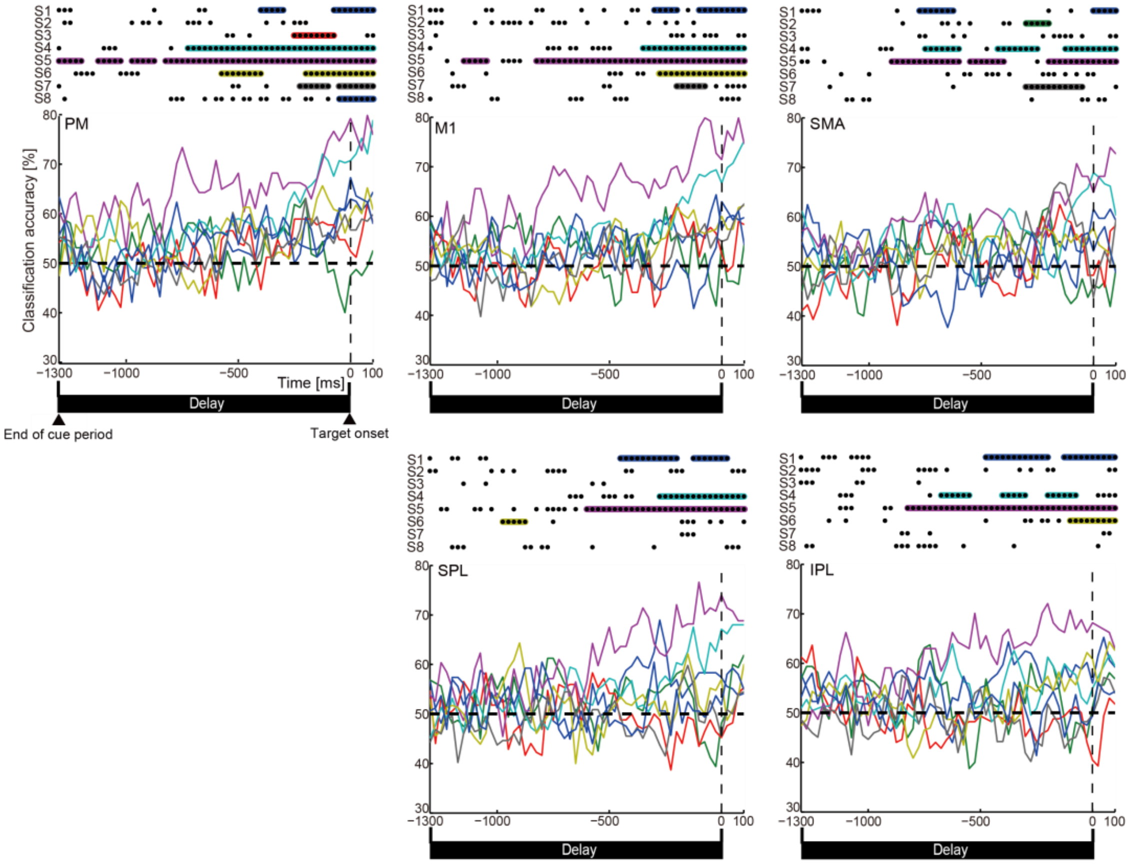

Supplementary Figure S3 | Classification accuracy of individual subjects in functionally selected areas. Time courses for individual subjects are color-coded. Rows of dots above each panel indicate significant time points (binomial test $p < 0.05$), and horizontal lines show consecutively significant time points corrected by a time-cluster-based approach (see Methods).

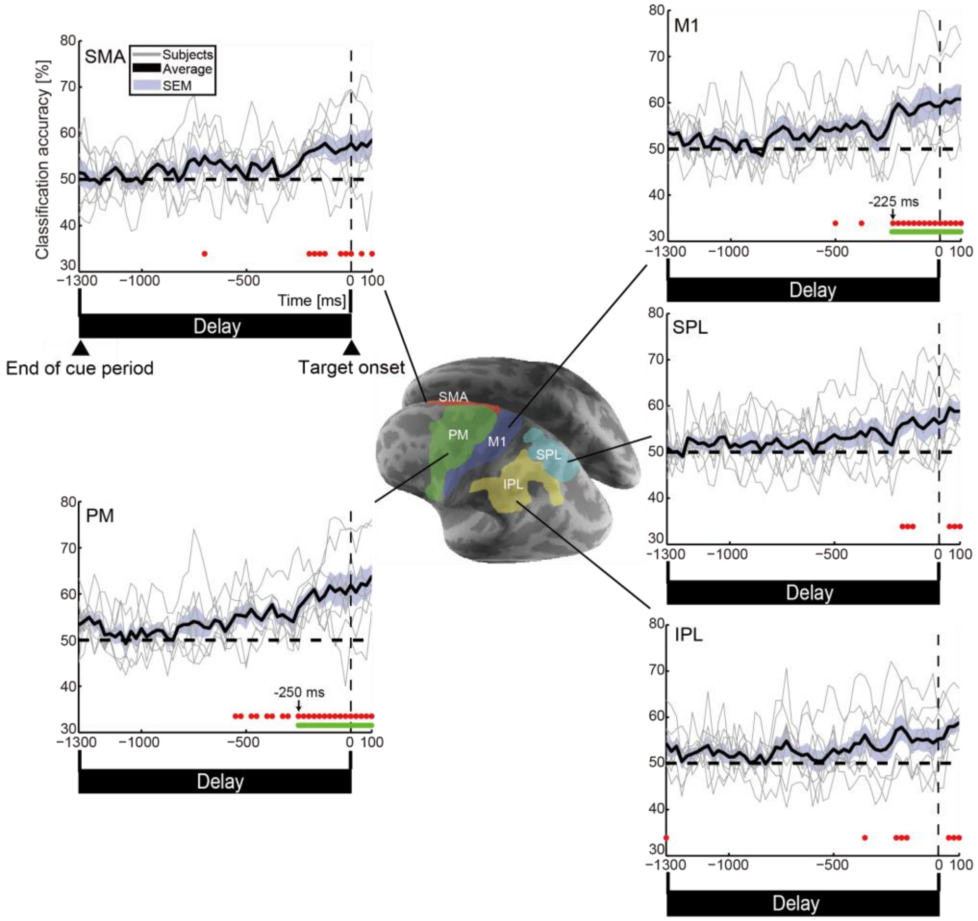

Supplementary Figure S4 | Classification results taking into account the influence of session-by-session differences in the number of short- and long-RT trials by incorporating a leave-one-session-out cross-validation procedure. We performed the classification analysis using the currents in left PM, M1, SMA, SPL and IPL from the start of the Delay period (-1300 ms) to 100 ms after target onset. Accuracies for individual subjects (thin gray solid lines) were averaged over all subjects (thick black solid lines). Blue shaded area denotes SEM across subjects. Rows of red dots indicate significant time points (n = 8, both two-tailed t-test $p < 0.05$ and group-level permutation test $p < 1\times10^{-4}$), and green horizontal lines show consecutively significant time points corrected by a time-cluster-based approach.

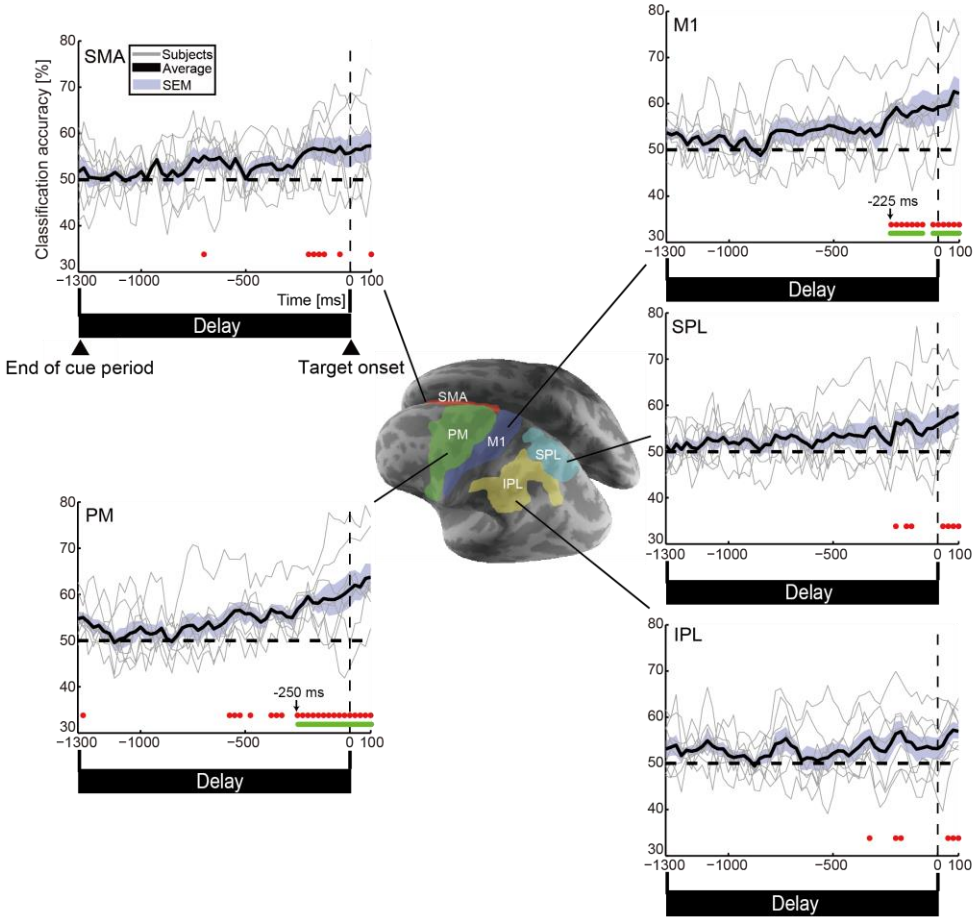

Supplementary Figure S5 | Classification results by equalizing the number of diploes across the motor-related regions by the random dipole-selection/equalization procedure (see main text). Accuracies for individual subjects (thin gray solid lines) were averaged over all subjects (thick black solid lines). Blue shaded area denotes SEM across subjects. Rows of red dots indicate significant time points (n = 8, both two-tailed t-test $p < 0.05$ and group-level permutation test $p < 1\times10^{-4}$), and green horizontal lines show consecutively significant time points corrected by a time-cluster-based approach.

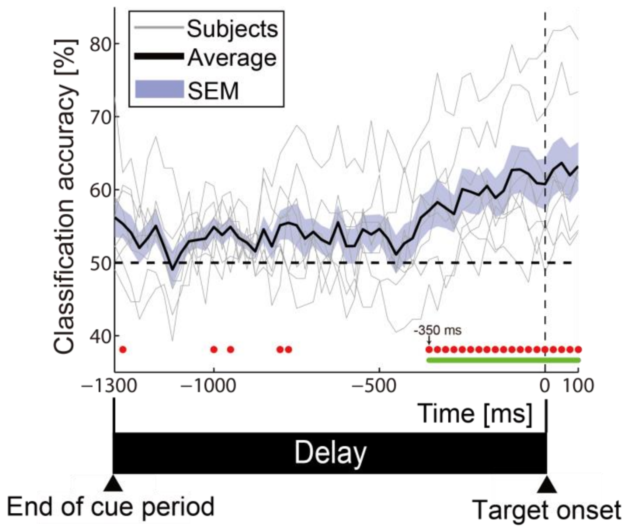

Supplementary Figure S6 | Classification result using MEG sensor signals. Accuracies for individual subjects (thin gray solid lines) were averaged over all subjects (thick black solid line). Blue shaded area denotes SEM across subjects. Rows of red dots indicate significant time points (n = 8, both two-tailed t-test $p < 0.05$ and group-level permutation test $p < 1\times10^{-4}$), and green horizontal lines show consecutively significant time points corrected by a time-cluster-based approach.

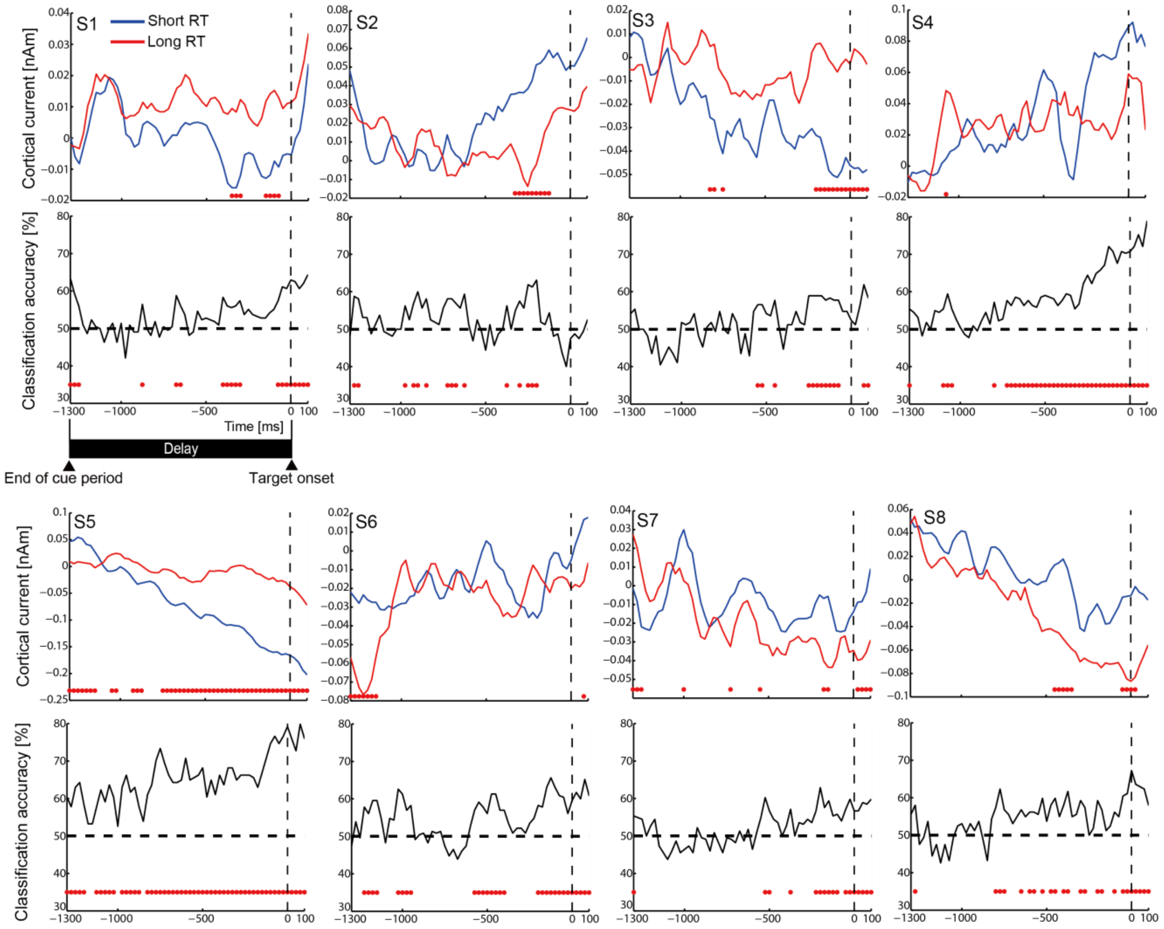

Supplementary Figure S7 | Comparison of within-subject cortical currents in short- with the long-RT trials. Panels in top and third rows show cortical currents averaged across dipoles in PM and trials within short-RT group (blue line) or long-RT group (red line). Trials were combined between the both fast- and slow-speed conditions. Rows of red dots indicate time points at which significant differences were identified (two-tailed *t*-test; $p < 0.05$). Panels in second and bottom rows indicate classification accuracy for each subject. Rows of red dots indicate time points of significant classification (binomial test; $p < 0.05$).

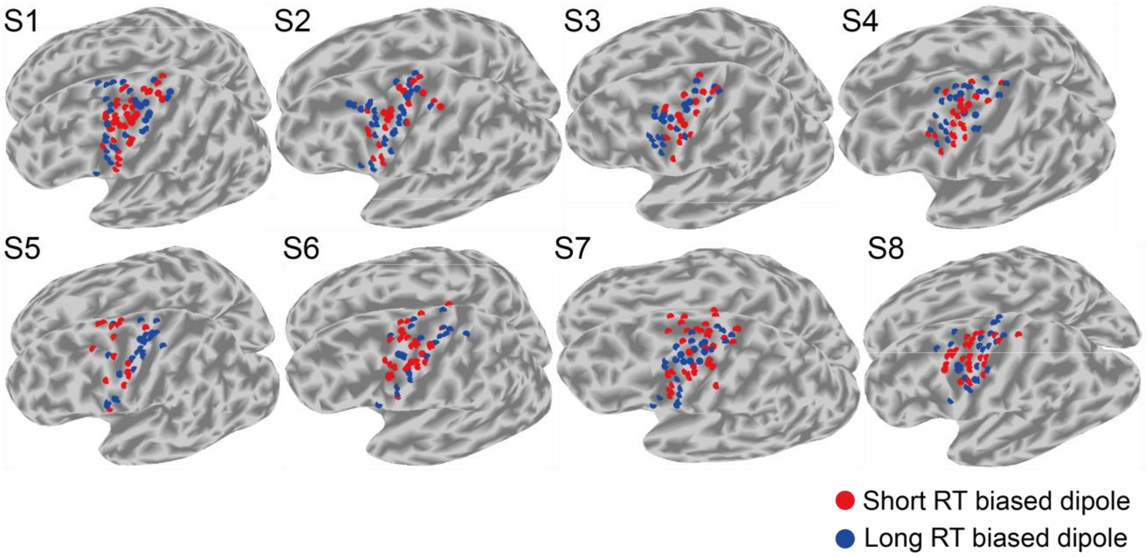

Supplementary Figure S8 | Dipole bias of classification at 50 ms before go-signal onset. Sign (positive: red or negative: blue) of product of weights and currents averaged across training data were plotted on PM's cortical surface. Positive or negative signs correspond to classification bias to short- or long-RT groups, respectively (see main text). We used weights and currents at 50 ms before go-signal onset, when significantly above-chance accuracy was obtained in most subjects.

SI Table

	S1	S2	S3	S4	S5	S6	S7	S8	Average	SD
PM	67	54	42	45	38	50	62	50	51.0	9.8
M1	33	30	38	37	31	37	38	30	34.3	3.6
SMA	26	21	25	21	27	18	22	27	23.4	3.3
SPL	38	38	37	35	40	38	35	21	35.3	6.0
IPL	32	54	40	30	47	55	46	41	43.1	9.2

Supplementary Table S1 | Number of dipoles in five motor-related regions for individual subjects (S1-8).

SI Text

Supplementary Methods

Feedback instruction

The feedback instructions were determined based on the maximum tangential velocity of the fingertips in each trial. The ideal maximum velocity was 75 and 55 cm/s in the fast- and slow-speed conditions. Subjects adjusted the maximum velocity within a range described below centered on ideal velocities. *Good*, *fast*, or *slow* were presented if the velocity was within, above, or below the range, respectively. The range was adjusted for individual subjects so that the probability of good instruction became about 50% (mean: 14.5 cm/s, SD: 1.4 across subjects). Subjects participated in practice sessions a few days before the MEG experiment for this adjustment to learn the ideal velocities.

Trial and sensor rejection criteria

Trials were excluded from analysis if individual data met at least one of the following criteria about behaviors, MEG, or EOG signals. For behavior criteria, 1) RT was less than 100 ms or the sum of the finger velocity sampled at 1 kHz during the Cue and Delay periods exceeded 10 cm/s, indicating subjects started too early (false start); 2) RT exceeded 1,000 ms or the sum of the finger velocity during the Move and Feedback

periods was less than 2 cm/s, indicating subjects started too late or didn't move; or 3) the distance between the target and the cursor was longer than half of the length from the fixation to the target. For the MEG signal criteria, the ratio of the maximum to the median signal values exceeded 10. Finally, for rejecting trials that included contaminated artifacts caused by eye movement and blinks, we set an EOG signal criteria whose signal value exceeded 40μA during the Cue, Move, and Feedback periods.

The sensor was considered impaired due to the instability of the MEG system if a time series of sensor signals (S) in a trial met the following criterion in more than 5% of the total trials: $\max(S_{ij})/\mathrm{median}(S_{ij}) > 10$ and $\max(S_{ij})/\mathrm{median}(S_{i*}) > 10$, where i and j are the indices of the trials and sensors and * means all of the sensors. All of the data obtained by that sensor were excluded from analysis.

Time-cluster-based approach for multiple comparison correction

The time-cluster-based approach is a correction method for multiple comparisons when multivariate pattern classification is applied to time-series data[23]. A time point was considered significant only when it was a member of a cluster of more than five consecutively significant time points. In our analysis, classification was repeatedly conducted using 100-ms sliding time window data in 25-ms steps. Thus, the classification

accuracies of adjacent time windows depend on each other (see red boxes in tw_i - tw_{i+3} in Supplementary Fig. S9). However, if the time windows are separated by more than five time points (see tw_i and tw_{i+4} in Supplementary Fig. S9), they are independent. Therefore, we used a cluster of five consecutively significant time points as the criteria for multiple comparison correction.

95% confidence interval estimation of onset of significant classification by a bootstrap method

We evaluated the 95% confidence intervals for the onset of consecutively significant classification[21, 24] for each of the five motor-related regions. We had the time courses of classification accuracy for eight subjects (thin gray lines in Fig. 3). A bootstrap sample consisted of eight time courses that were randomly selected from the eight subjects with replacements (i.e., the same subject's time-course was allowed to be selected multiple times). Both a two-tailed *t*-test and a group-level permutation test were performed for the bootstrap sample to determine the onset of consecutively significant time points, as in the original analysis (see Methods). This procedure was repeated 1,000 times to obtain 1,000 bootstrap estimates of onsets, resulting in the determination of 95% confidence intervals.

Classification method based on a leave-one-session-out cross-validation procedure

To investigate whether the classification results were influenced by the session-by-session difference in number of trials belonging to short- and long-RT groups, we incorporated a leave-one-session-out cross-validation procedure in our classification method. First, the classifier was trained using trials in the fast-speed condition except for one session's data (e.g. the second to the eighth session data in fast-speed condition at #1 in Supplementary Fig. S10), and we then evaluated classification accuracy using the one left-out session's data in slow-speed condition (e.g. the first session data in slow-speed condition at #1 in Supplementary Fig. S10). We repeated this procedure until all sessions' data in slow-speed condition became test data. We also carried out classification in the reverse direction, that is, the classifier was trained by slow-speed condition data and tested by fast-speed condition data, and then estimated the averaged decoding accuracy.

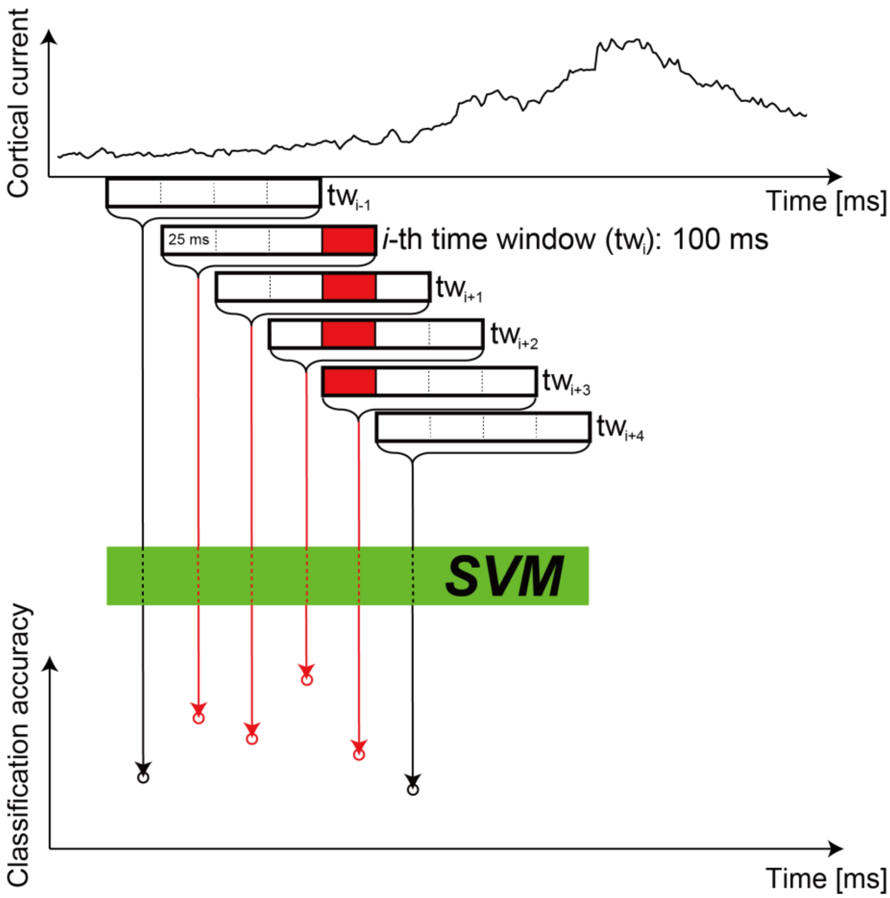

Supplementary Figure S9 | Schematic of sliding time window for decoding analysis. Cortical currents temporally averaged within 100-ms time windows were used as SVM classifier's input data. Time window slides were in 25-ms steps. Shared data at time windows (red boxes in tw_i - tw_{i+3}) does not affect accuracy at time windows after four moving steps (tw_{i+4}).

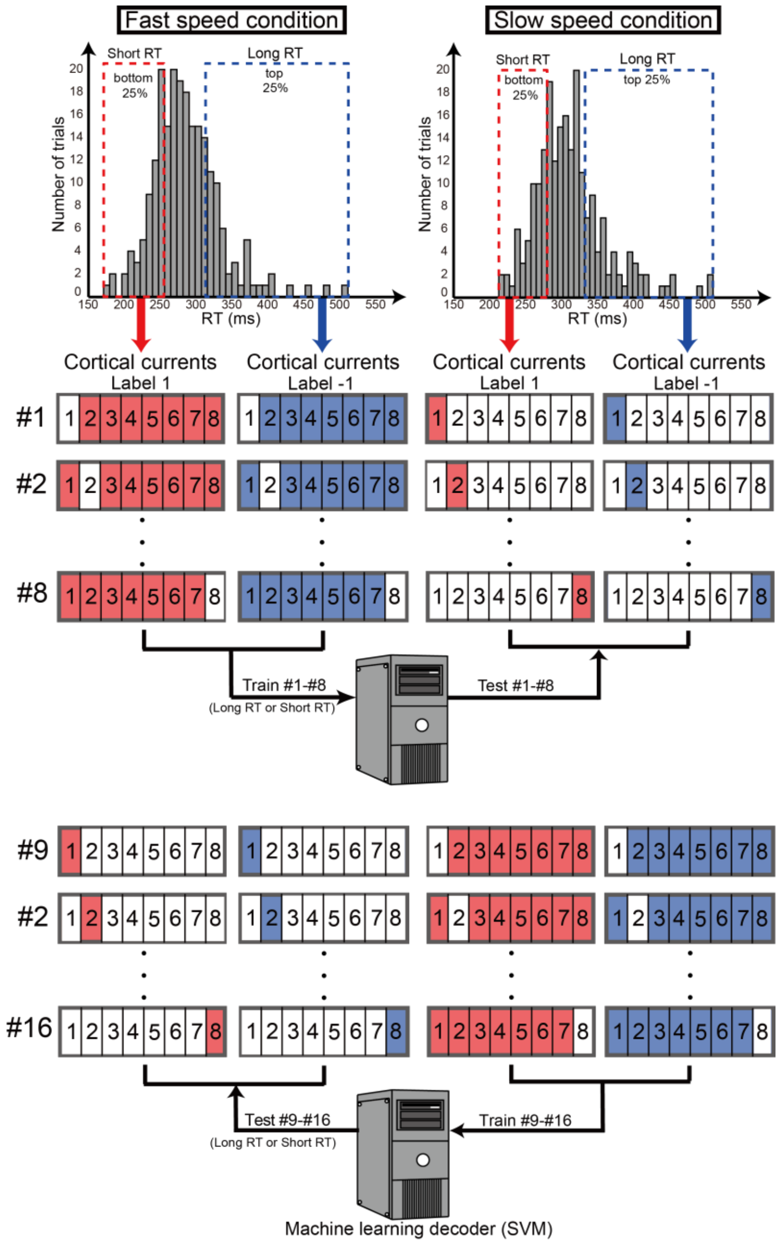

Supplementary Figure S10 | Schematic illustration of classification analysis based on a leave-one-session-out cross-validation procedure. Machine learning decoder was trained using trials in one speed condition without one session's data and tested by the left-out session's data in the other speed condition. This procedure was repeated until all sessions' data in both speed conditions became test data. For instance, Subject 1 underwent eight sessions in the MEG experiment, and thus we repeated the procedure 16 times (16 validation folds). # indicates the number of the validation fold. The number inside the boxes indicates session number, and the boxes colored by red and blue indicate the cortical currents in short- and long-RT trials, respectively, in training and test data.
